# Spatial distribution of water reservoirs in the Sota catchment (Benin, West Africa) and implications for local development

**DOI:** 10.1016/j.heliyon.2023.e14458

**Published:** 2023-03-11

**Authors:** Kevin S. Sambieni, Fabien C.C. Hountondji, Luc O. Sintondji, Nicola Fohrer

**Affiliations:** a Graduate Research Program on Climate Change and Water Resources/West African Science Service Center on Climate Change and Adapted Land Use, University of Abomey-Calavi, Benin; b Faculté d’Agronomie, Université de Parakou, Parakou, Benin; c Institut National de l’Eau, Université d’Abomey-Calavi, Abomey-Calavi, Benin; d Department of Hydrology and Water Resources Management, Institute for Natural Resource Conservation, Kiel University, Germany

**Keywords:** Basin management, Geology, Livestock, Population, Rainfall, Spatial distribution, Sota catchment, Water reservoirs

## Abstract

Water is an indispensable resource for human being and the environment. This study analyses spatial distribution of water reservoirs in the Sota catchment with regards to livestock density, population density, rainfall distribution and geological structure of the Sota catchment, and assessed the state of these reservoirs. To this end, the geographic coordinates of water reservoirs were collected and the updated database of reservoirs census in Benin, was used. In addition, livestock and population census database, rainfall data from 1980 to 2016 of twelve (12) stations and geological database were processed in ArcGIS for generating respectively the spatial layers of livestock, population, rainfall and geological map of the catchment. The reservoirs' state has been appreciated by documents exploration and in situ observations. Single factor Regression analysis was conducted to understand the relation of each of the factors with the spatial distribution of the reservoirs in the Sota catchment. The results reveal that Sota catchment contains 35 small water reservoirs mostly concentrated in its central western and south western part. The reservoir density is 0.0026 km^−2^. Most of the reservoirs are located within areas where livestock density, population density and rainfall amount is high: 51%, 46% and 86% respectively. However, no significant relation was found between reservoirs distribution and livestock density, population density, and rainfall respectively in the catchment. The basement geological structures of the Sota catchment are associated with 71% of the reservoirs' location. The reservoirs are threatened by siltation, lack of pastoral facilities, poor maintenance and management. In fact, 100%, 86%, 74%, 71%, 40%, and 34% of reservoirs are respectively subjected to the issues of: absence of waterers, siltation, deteriorated dyke, eutrophication, inexistent management committee, and drying up in dry season. For sustainable local development purposes, more attention should be paid to basin management planning for construction of new reservoirs and to reservoirs ‘maintenance. Future research on the reservoirs’ sustainability and monitoring surveillance are recommended.

## 1 Introduction

Water resources are required by all living creatures and societies for survival [1] but are seriously affected by climate change. Indeed, since 1970 the Sub-Saharan region in general, has been experiencing strong spatial and temporal rainfall variability as a result of climate change, with huge impacts on water resources [2,3]. The severe droughts of the 1970s in the Sahelian countries, with severe water shortage and starvation, compelled donors and governments to promote reservoirs to water livestock and to enhance irrigated cereal production; in brief, to enhance food security [4,5]. In West Africa, the construction of reservoirs is found to be a sound strategy in assisting people to adapt to climate change, to increase water storage capacities, regulate water flows, contribute to food security, increase livelihood resilience, and maintain or/and improve wetland ecosystem functions and services [4,[6], [7], [8]]. Water storage is an important tool for resilience [9] but reservoirs as an important human activity also affect runoff [10], environment, public health [11] and lead to water loss by evaporation [[12], [13], [14]].

In Benin, a West African country, the 1970s droughts caused high mortality of ruminants (cattle, sheep, and goats) especially in its northern part [15]. To face these challenges, the government of Benin initiated the construction of reservoirs in the northern part of the country [15,16]. It appears that the majority of water reservoirs in Benin have been implemented in the districts included in the Benin portion of the Niger River Basin (BPNRB) [17] which is an agro pastoral basin [18]. The reservoirs constructed, are used as multi-purpose facilities such as livestock watering place, fish farming, domestic purposes, vegetable production, food cropping, washing, swimming, small business water use, brick making, house and road construction [16,19]. These reservoirs have therefore become vital assets in local people’s livelihood [20]. As a result, given their importance, the reservoirs have been the target of many studies in Benin. These studies mostly focused on reservoirs management [19], reservoirs siltation [15] and reservoirs water quality [[21], [22], [23]]. Little is known about their spatial distribution. That implies our limited knowledge on that matter. Moreover, these studies were not carried out at the basin scale but at the administrative boundaries. Yet basins are the natural unit of area in which water resources can be well managed. This study sought to fill these gaps by investigating the spatial distribution of water reservoirs in the Sota catchment, a sub basin of the Benin portion of the Niger River Basin. A better understanding of the spatial distribution of water reservoirs ensures equitable and accurate water management interventions for local development, since the scattering of reservoirs contributes considerably to the food security and livelihoods [11]. We hypothesize that population density, livestock density, rainfall and geology are valuable factors to significantly explain the spatial distribution of water reservoirs in the Sota catchment. In the other hand we hypothesize that the reservoirs are not in good state.

This paper first analyses the spatial distribution of the reservoirs in the Sota catchment, with respect to livestock density, population density, spatial rainfall distribution and geology. Then, the state of the reservoirs is appreciated and at last, the implications of spatial distribution of reservoirs for local development is discussed.

## 2 Materials and methods

### 2.1 Study area

**Fig. 1:**
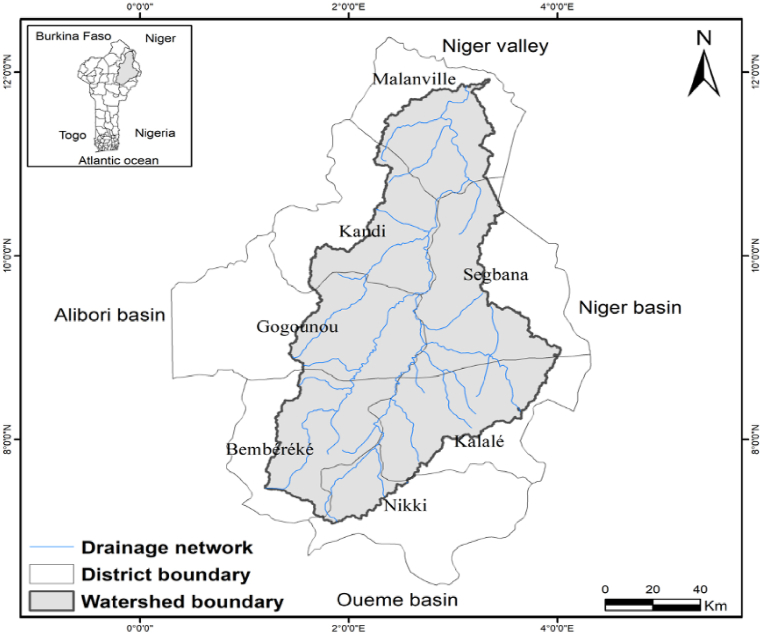
Map of the Sota catchment.

**Fig. 2:**
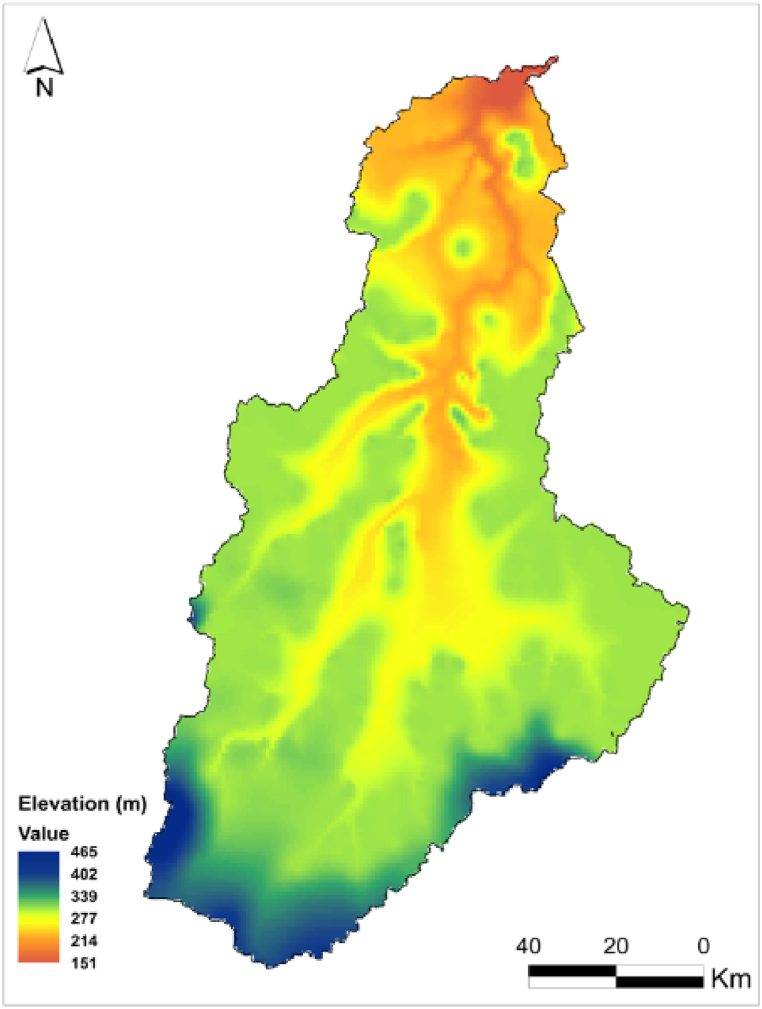
DEM map of the Sota catchment (30 m resolution SRTM).

The area under study is the Sota catchment, a sub-basin of the Benin portion of the Niger River Basin (BPNRB) located in the Northern part of Benin. Sota catchment is located between 9°54′ and 11°45′ north latitude and between 2°28′ and 3°52′ east longitude (Fig. 1). Its surface area covers 13,410 km^2^, i.e. 11% of that of Benin, and includes the sub-basins of Sota at the outlet of Gbassè (8300 km^2^) and the sub basin of Sota at the outlet of Coubéri (13,410 km^2^). The Sota catchment is shared by seven districts namely: Malanville, Ségbana, Kandi, Gogounou, Bembèrèkè, Kalalé, and Nikki (Fig. 1). The hydrographic network in the Sota catchment area consists of the Sota River (which is 254 km long) and its tributaries (Fig. 1). Climate of the basin is tropical with a unimodal rainfall. Average annual rainfall ranges from 700 mm (Sudan savannah) to 1000 mm (Guinea savannah). Monthly average temperature varies between 24.9 °C and 32.5 °C, while the annual average revolves around 27.9 °C [11]. The relief in the Sota catchment area is generally inclined from south to north with altitudes varying between 151 m and 465 m (Fig. 2). The main economic activities of local populations in the catchment are: slash and burn agriculture, livestock raising, wood energy production, forest logging, and fishing. The main crops are cotton, maize, sorghum and rice [24,25]. Secondary crops include groundnuts, beans, yams cassava, sweet potatoes, and millet. Cotton is the main cash crop [24,25]. Livestock production is the second largest economic activity in the basin after crop production [25]. Land cover around the reservoirs is the result of agricultural activities around the reservoirs and grazing of surrounding pasturelands. The local stakeholders involved in the reservoirs belong to different ethnic groups. Local and transhumant herders, for instance, belong to the Peul ethnic group, therefore livestock watering at the reservoirs is mostly carried out by this ethnic group whereas the other stakeholders are of mixed ethnicity (Bariba, Dendi, etc …) and use the reservoirs for fishing, vegetable production, domestic uses etc … [19].

### 2.2 Spatial distribution of water reservoirs

Spatial distribution analysis requires the collection of water reservoirs coordinates. To this end, registers and inventories (reservoirs, etc.) developed by national institutions, mainly the Directorate General of water (DGEau), the Territorial Agencies for Agricultural Development (ATDA), have been used. The Directorate General of water (DGEau) carried out in 2019 the most inventory of water reservoirs in Benin. The DGEau data [26] are presented as a series of attributes (reservoirs coordinates, survey date, location, administrative features, state of dykes, storage capacity, date of construction, water uses, siltation state, eutrophication, etc …). Apart from the DGEau data, field data were collected in the study area in the dry season (from December 2020–February 2021) and in the rainy season (from July 2021 to September 2021) in order to check the reservoirs' location and get coordinates of reservoirs not included in the DGEau data and additional information on reservoirs' state. All the data collected were checked (quality, mode of acquisition, comparison of sources), treated (reformatting and organization) and validated. By merging information obtained through the census of the reservoirs provided by DGEau and field work, we obtained water reservoirs' database used for this study.

Then, the ArcGIS software was used to incorporate all collected information and to generate maps. To map the spatial distribution of the reservoirs in the Sota catchment, water reservoirs coordinates were projected in the first Northern UTM zone (zone 31N) projection, based on the WGS 84 datum. Our data are all concentrated in this zone, and WGS 84 is the datum used by the global positioning system (GPS). Indeed, to make sure that all the reservoirs of the Sota catchment have been taken into account, all the registered points of the database were projected in the UTM zone 31N, WGS 84. Then, the shapefile of the Sota catchment (representing the limit of the Sota catchment) has been also projected. The points which fell into the limit of the Sota catchment have been recorded and those aside the limit of the Sota catchment have been excluded.

### 2.3 Factors of water reservoirs' distribution

#### 2.3.1 Livestock distribution

To collect data on livestock size in the Sota catchment, the database of the most recent livestock inventory carried out in 2015 in Benin, was used. This database [27] was obtained from the Ministry of agriculture, breeding and fisheries of Benin. Then livestock size of the different districts included in the Sota catchment has been extracted from this database. Livestock density of the different districts included in the Sota catchment was calculated by dividing livestock size of each district by its surface area. Then, livestock density of each district was incorporated and processed in ArcGIS software to generate the spatial layer of livestock density in the whole area of the districts sharing the Sota catchment.

#### 2.3.2 Demography

To have the size of the population in the Sota catchment, the most recent database of the national census of the population in Benin [28] carried out in 2013, was used. This database was obtained from the National Institute of Statistics and Applied Economy of Benin (INSAE). The population size of the seven (07) districts included in the Sota catchment has been extracted from this database. Population density of the different districts included in the Sota catchment was calculated by dividing population size of each district by its surface area. Then, population density of each district was incorporated and processed in ArcGIS software to generate the spatial layer of population density in the whole area of the districts sharing the Sota catchment.

#### 2.3.3 Climate and geology

Climate is an important natural factor of reservoir’s distribution, especially rainfall. A total of twelve rainfall stations were identified of which seven stations inside the Sota catchment and five stations around the catchment. Yearly rainfall data of these stations available from 1980 to 2016, were collected at Benin Meteorological Agency. Then, these data were interpolated using ordinary Kriging method in ArcGIS to generate the map of the spatial distribution of rainfall in the Sota catchment. In fact, spatially distributed rainfall can be interpolated by a range of different methods but no one interpolation method stands out as being universally the best. The reason of applying ordinary kriging methods is that Kriging presents an important advantage in its ability to take into account the spatial correlation between the data recorded at different rain gauges or weather stations and give unbiased predictions with minimum variance [29].

Geology is also an important natural factor of water reservoirs' distribution in a given area. The database on the geology of Benin was obtained from the «Office Beninois des Mines». This database [30], was incorporated and processed in ArcGIS to extract the geological map of the Sota catchment.

### 2.4 State of the reservoirs

The state of the water reservoirs was evaluated on the one hand by exploring DGEau database [26] and on the other hand during the field work mainly by direct observation. The following parameters were assessed: state of the dyke, presence of waterer for livestock watering, reservoirs' eutrophication, siltation, reservoir drying up, existence of reservoir management committee, activities related to the reservoirs. Regarding the state of dyke, we considered as deteriorated dyke, any dyke with apparent cracks, invaded by grasses and shrubs. Reservoirs subjected to eutrophication were identified by observing the accumulation of algae on the water surface and the presence of croplands near the reservoirs.

### 2.5 Statistical analysis

A simple linear regression analysis was applied in order to determine if there is a significant correlation between reservoirs' distribution and livestock density, population density and annual average rainfall in the Sota catchment. To this end the number of reservoirs per district of the Sota catchment was taken as the dependent variable and the independent variables considered are: livestock density, population density and annual rainfall amount. Each independent variable was taken separately and analyzed with the dependent variable. The regression analysis is based on the equation:

(1) Y = β_0_ + β_1_X + ε

where Y is the dependent variable, X the independent variable, β_0_ is the intercept (the predicted value of Y when the X is 0), β_1_ is the slope, and ε is the error of the estimate. The null hypothesis H_0_ is that there is no association between the dependent variable and the independent variable (H_0_: β1 = 0) and the alternative hypothesis H_A_ is that there is an association between the dependent and independent variables (H_A_: β1#0). The regression analysis was performed using R software.

## 3 Results

### 3.1 Spatial distribution of water reservoirs in the Sota catchment

**Fig. 3:**
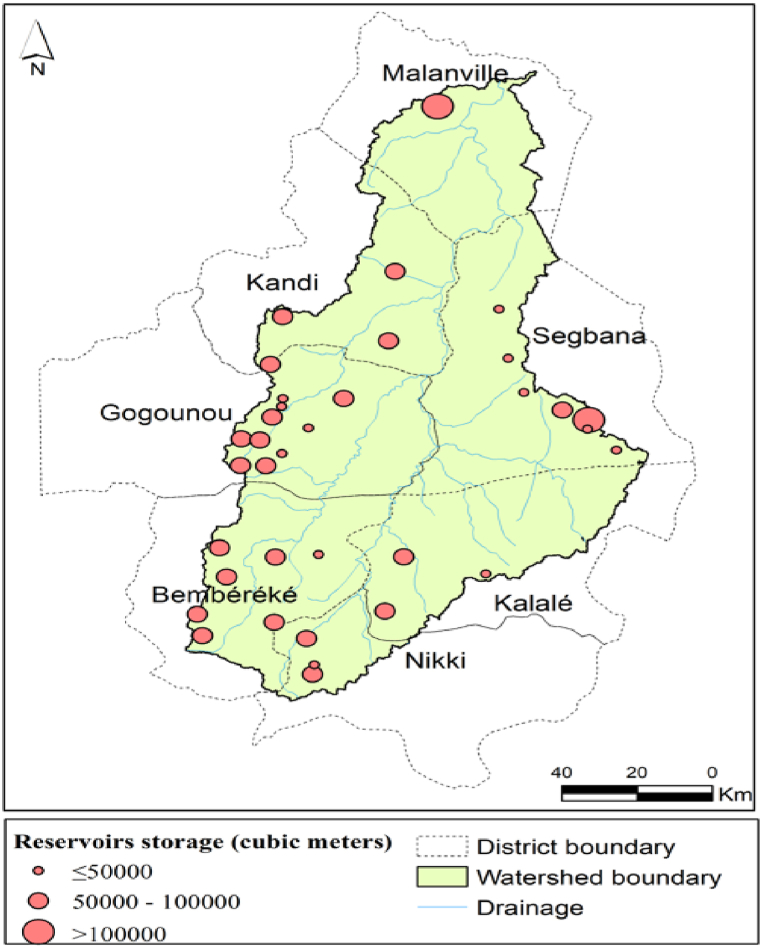
Water reservoirs' sites in the Sota catchment.

Fig. 3 shows the location of water reservoirs in the Sota catchment.

Fig. 3 indicates that the Sota catchment is home to 35 water reservoirs scattered all over the catchment. These reservoirs are more concentrated in the central western part and south western part of the catchment. The northern part of the Sota catchment appears to be the least supplied with water reservoirs. The reservoir density can be estimated at 0.0026 km^−2^ (2.6/1000 km^2^).

Reservoirs which storage capacity is between 50,000 m^3^ and 100,000 m^3^, are the most dominant in the Sota catchment. The smallest reservoir has a storage capacity of 10,000 m^3^ and is located in the district of Nikki. The biggest reservoir with a storage capacity of 180,000 m^3^ is located in the district of Malanville. According to the International Committee On Large Dams [31] standards, all the reservoirs in the Sota catchment can be qualified as “**small reservoirs**”, since these are engineered surface water bodies with a capacity of less than one million m^3^ (Mm^3^).

**Fig. 4:**
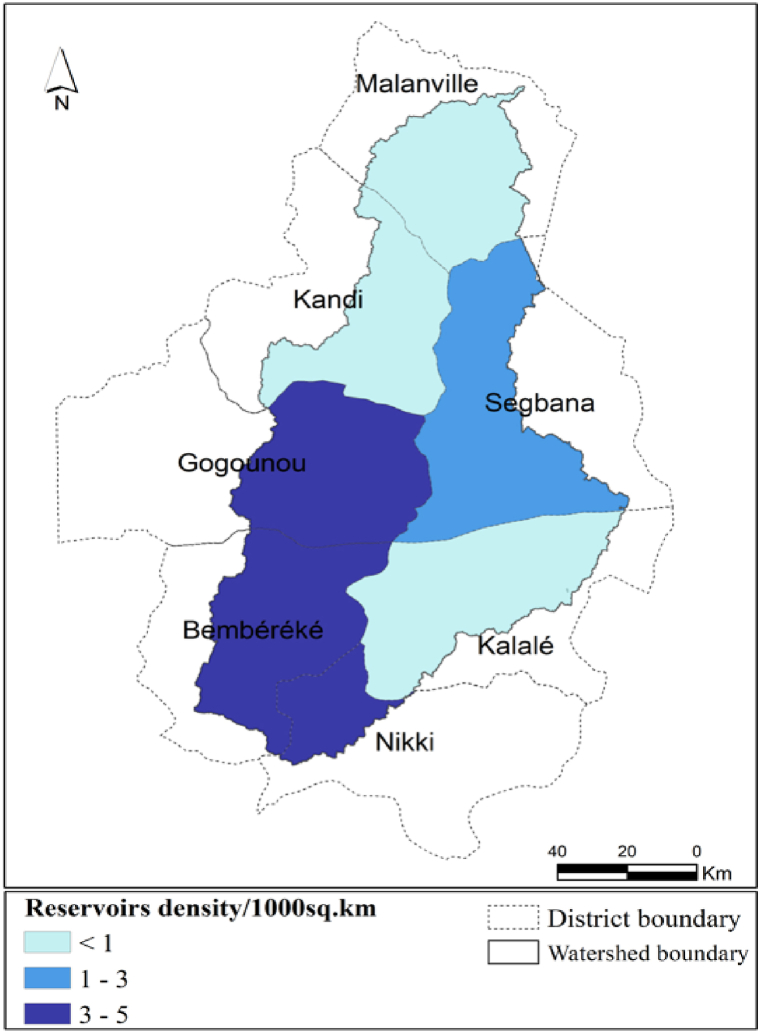
Reservoirs density distribution per district in the Sota catchment.

Fig. 4 presents the distribution of reservoirs in the Sota catchment, in terms of density per district.

Fig. 4 highlights the reservoirs density in the different sections of the districts sharing the Sota catchment. The analysis of Fig. 4 reveals that the portion of the districts of Gogounou, Bembereke and Nikki in the Sota catchment, have the highest reservoirs density varying from 3 to 5 reservoirs per km^2^. The lowest reservoirs density is observed in the Northen part and the South Eastern part of the Sota catchment namely in the portion of the districts of, Malanville, Kandi and Kalale.

**Fig. 5:**
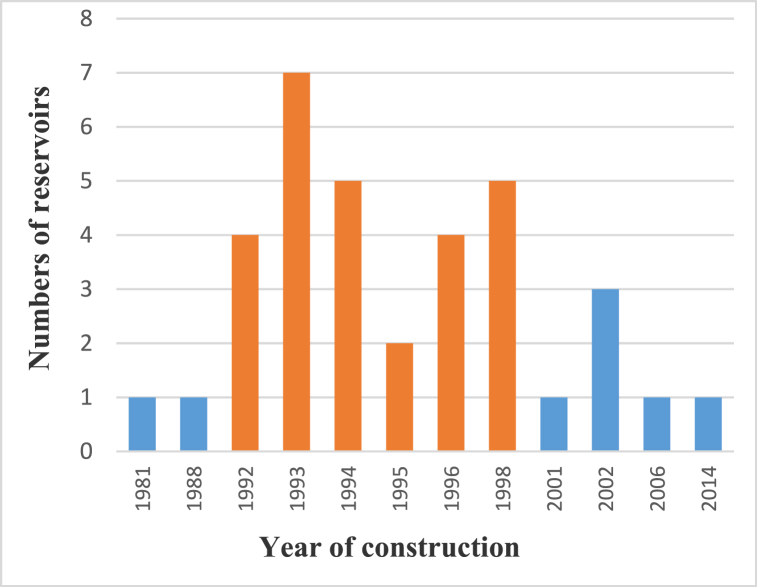
Timeline of construction of the reservoirs in the Sota catchment.

The reservoirs were built at different times. Fig. 5 shows the rate of implementation of water reservoirs in the Sota catchment over time.

Fig. 5 indicates that the oldest reservoir in the Sota catchment was built in 1981 and most of the reservoirs were built after the 1990s. In fact, out of the 35 reservoirs in the Sota catchment, 27 (77%) reservoirs were built in the seven years between 1992 and 1998 (indicated in the orange color in Fig. 5). This corresponds to the period following the drought crisis of 1970s and 1980s occurred in Benin and in the West African countries causing high livestock mortality and starvation [4,5,19,32].

The authorities having seen the damages caused by these droughts, engaged to secure livestock and people from future droughts. The reservoirs, not only in the Sota catchment but also in the whole Benin, were constructed through Government, NGOs and international cooperation initiatives [15,19]. The Agro Pastoral Hydraulics Program of Benin (PHPA) is a key example of these. The reservoirs are constructed in most cases from earth materials and according to Ref. [17] their dissemination in Benin depends on the size and the distribution of livestock and the need for water.

### 3.2 Distribution of the reservoirs with respect to livestock density, population density and rainfall distribution in the Sota catchment

The reservoirs serve mainly for livestock watering and then for other needs: fish farming, vegetable production, domestic uses etc … [26]. Livestock is mainly composed of oxen and few sheep. Table 1 shows livestock size, population size, average annual rainfall (1980–2016) for each district located in the Sota catchment.

**Table 1:** Number of water reservoirs, livestock size, human population and average annual rainfall (1980–2016) per district sharing the Sota catchment.

Districts	Livestock size (MAEP, 2015)	Population size (RGPH 4, 2013)	Average annual rainfall (1980–2016) (mm)
Malanville Kandi Segbana Gogounou Kalale Bembereke Nikki	69,770 163,130 77,260 142,610 176,470 130,300 128,250	168,641 179,290 89,081 117,523 168,882 131,255 151,232	784.26 990.88 1013.4 990.88 1063.35 1085.13 1054.27
**Total**	**887,790**	**1,005,904**	

Livestock size and population size presented in Table 1 are not just limited to the Sota catchment area but these are global data covering the whole area of the districts sharing the Sota catchment.

**Fig. 6:**
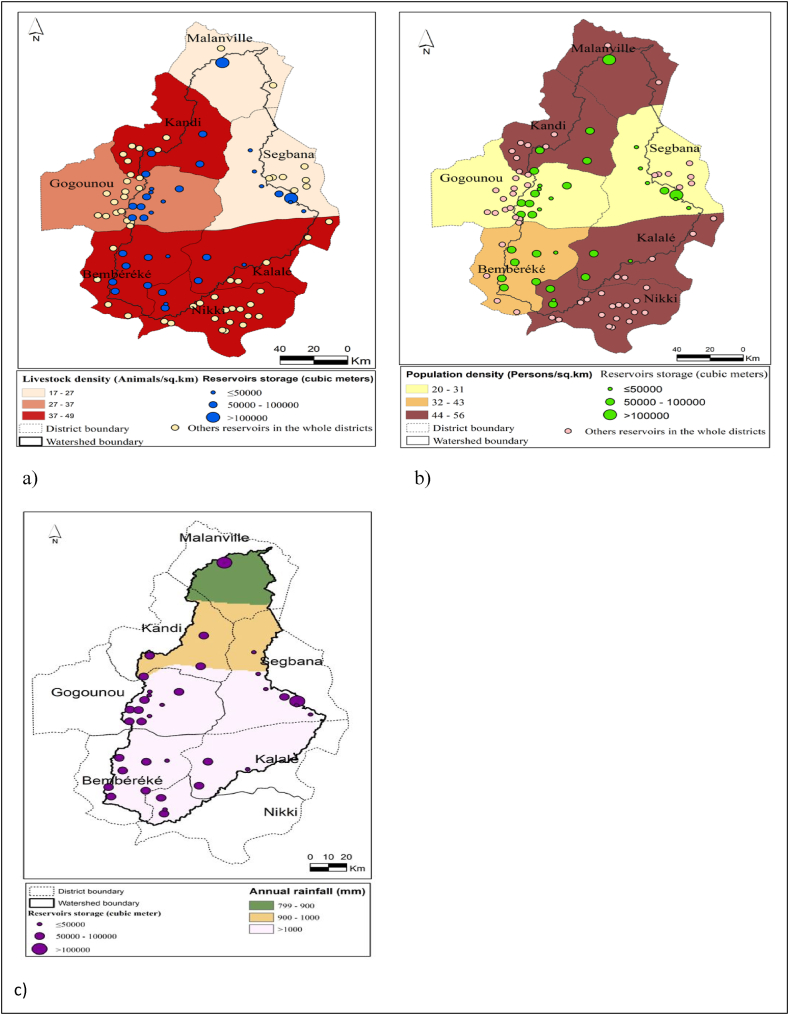
Spatial distribution of water reservoirs with respect to a) livestock density in the whole area of the districts sharing the Sota catchment; b) population density in the whole area of the districts sharing the Sota catchment and c) rainfall distribution in the Sota catchment.

The spatial distribution of water reservoirs with respect to livestock density, population density and rainfall distribution in the Sota catchment is presented in Fig. 6. Since global livestock and population size data at the district were used for livestock and population densities calculation and mapping, all the reservoirs included not only in the Sota catchment but in the whole area of the districts sharing the Sota catchment, were projected and took into account to make a good interpretations of the reservoirs distribution according to livestock density, and population density.

Fig. 6a indicates that livestock is more concentrated in the southern part and the north western part of the Sota catchment. In addition, most of the reservoirs are located in the high livestock density areas namely Bembereke, Kalale, Kandi, and Nikki. In fact, 56% of the reservoirs are located in the high livestock density areas whereas 26% and 18% of the reservoirs are located respectively in the medium and low livestock density areas.

In terms of reservoir storage, the medium storage capacity (50,000–100,000 m^3^), is dominant and more associated with high livestock density areas within the Sota catchment namely: Bembereke, Nikki, Kalale, Kandi, and Gogounou.

The analysis of Fig. 6b reveals that population is more concentrated in the south eastern part, and in the north western part of the Sota catchment. It is observed that most of the reservoirs are located in the high population density areas. In fact 46% of the reservoirs are located within areas where population density is high (Nikki, Kalale, Kandi and Malanville), whereas 41% and 13% of the reservoirs are located respectively within areas of low and medium population density. The biggest reservoir is located in a high population density area namely Malanville with a storage capacity of 180,000 m^3^. Nevertheless it is important to highlight the high rate (41%) of reservoirs in the lowest population density areas namely Gogounou and Segbana. More, the district of Malanville having high population density area has only three reservoirs representing the lowest number of reservoirs per district.

The analysis of Fig. 6c reveals that the northern part of the Sota catchment is prone to lower rainfall amount compared to the central and southern part. 86% of the total number are located in the high rainfall amount areas. 11% and 0.035% of reservoirs are located respectively within areas where annual rainfall amount is moderate and low. Therefore, most of the reservoirs in the Sota catchment are located in the area prone to high rainfall amount. The biggest reservoir is located in the low rainfall amount area namely the district of Malanville.

### 3.3 Correlation between water reservoirs and livestock density, population density, and rainfall in the Sota catchment

**Fig. 7:**
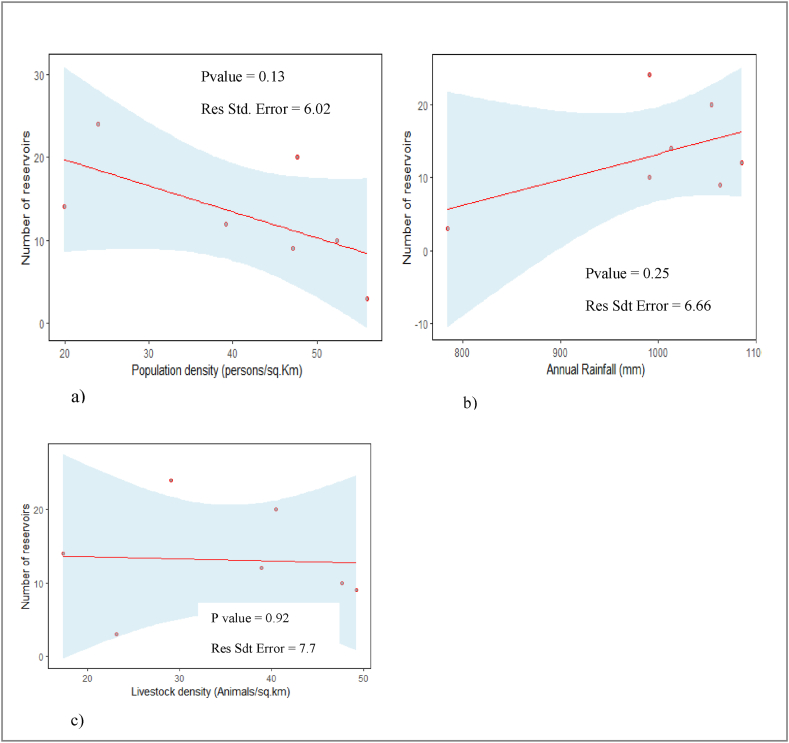
Correlation between number of reservoirs and a) population density; b) average annual rainfall c) livestock density in the Sota catchment.

The previous point highlighted how water reservoirs are distributed with regards to livestock density, population density and rainfall distribution in the Sota catchment. Do these factors significantly explain the spatial distribution of water reservoirs in the Sota catchment? The results from the simple regression analysis applied between number of reservoirs and livestock density, population density and rainfall amount of each district are displayed in Fig. 7.

Fig. 7 indicates that there is no significant correlation between the number of reservoirs with population density, livestock density and annual rainfall in the Sota catchment, all three giving a *p*-value > 0.05. As a result, neither population density nor livestock density and rainfall, significantly explain the spatial distribution of reservoirs within the Sota catchment. It is important to highlight that our sample size including only seven (7) observations which represent the seven districts, is small to make a strong regression analysis. In fact a sample size of 30 observations is more appropriate for a strong regression analysis.

### 3.4 Spatial distribution of reservoirs with overlaying of geological structures of the Sota catchment

**Fig. 8:**
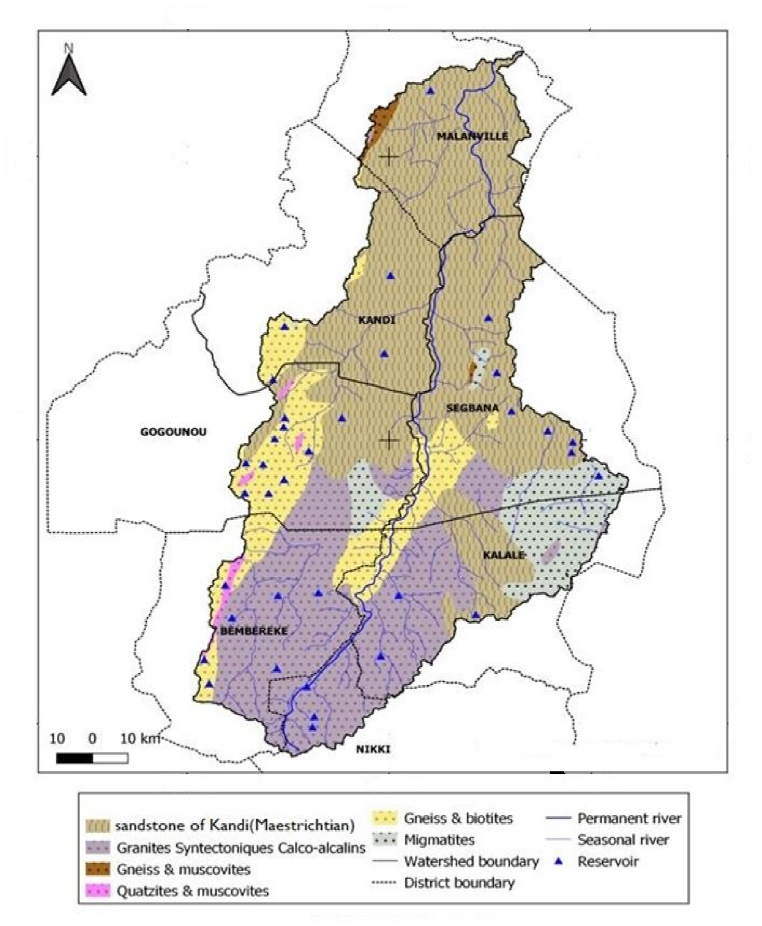
Reservoirs' distribution associated with geological structures of the Sota catchment.

Regarding the link between reservoirs and geology, the geological structures have been overlaid with the reservoirs location to identify the geological formations associated to the reservoirs. Fig. 8 represents the reservoirs map with overlaying of geological structures of the Sota catchment.

From a general point of view the geological structure of the Sota catchment can be divided into two distinct geological formations from south to north: the basement structures in the south and the sedimentary sandstone structures of Kandi in the north [25,33,34].

The basement structure comprises essentially gneiss, biotite, granite, muscovite and migmatite, quartzite. The basement structure is impermeable below a considerable alteration zone. But the different sedimentary sandstone facies have (due to their porosity) a high degree of permeability and they have significant storage capacity [24,25]. Their aquifer potential is thus relatively important [34,35]. The analysis of Fig. 8 reveals the reservoirs' location in Sota catchment is mostly associated with the geological basement structures. In fact, 71% of the reservoirs are located in geological basement structures and 29% in the geological sedimentary structures.

In terms of drinking water supply, groundwater resources are the main source of drinkable water for population in the Sota basin. Ref. [25] reported that drinking water is not a major issue in the cities of the basin. The districts benefit from groundwater infrastructures (boreholes and large diameter wells) implemented by «Société Nationale des Eaux du Bénin (SONEB)» and «Direction Générale de l’Eau» [25].

### 3.5 Reservoirs' state of the Sota catchment

**Fig. 9:**
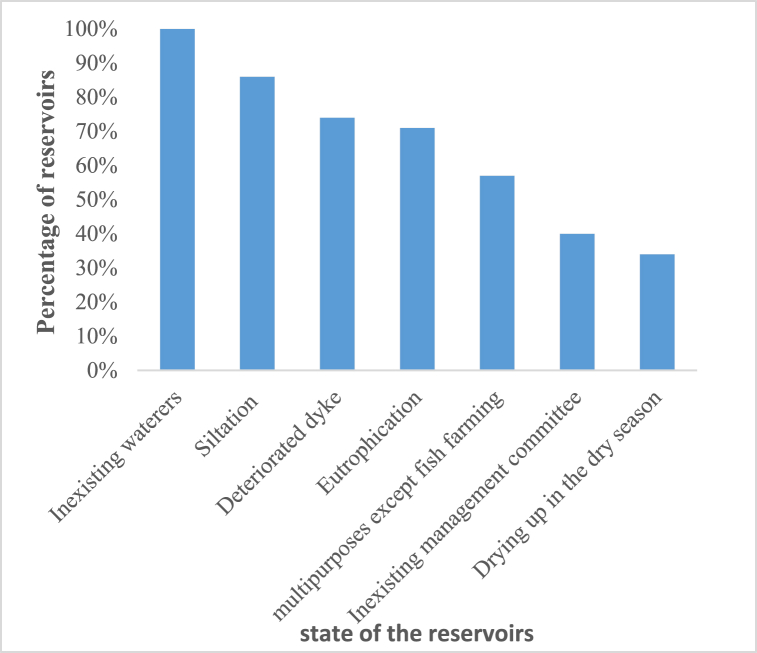
Percentage reservoirs affected by potential deteriorating factors in the Sota catchment.

Fig. 9 presents the percentage of reservoirs affected according to potential deteriorating factors.

The analysis of Fig. 9 reveals that the reservoirs are subjected to potential deteriorating factors i.e. absence of waterers for livestock watering, siltation, deteriorated dyke, eutrophication, multipurpose uses except fish farming, poor management (lack of management), and drought. Siltation issue observed at 84% of the reservoirs, reduces their water storage and increases their vulnerability to early dry up. Agricultural intensification, population growth and urban expansion are likely to make the reservoirs more eutrophicated and silted. Drying up of reservoirs, is observed in the months of March and April and hinders the use of the reservoirs.

**Fig. 10:**
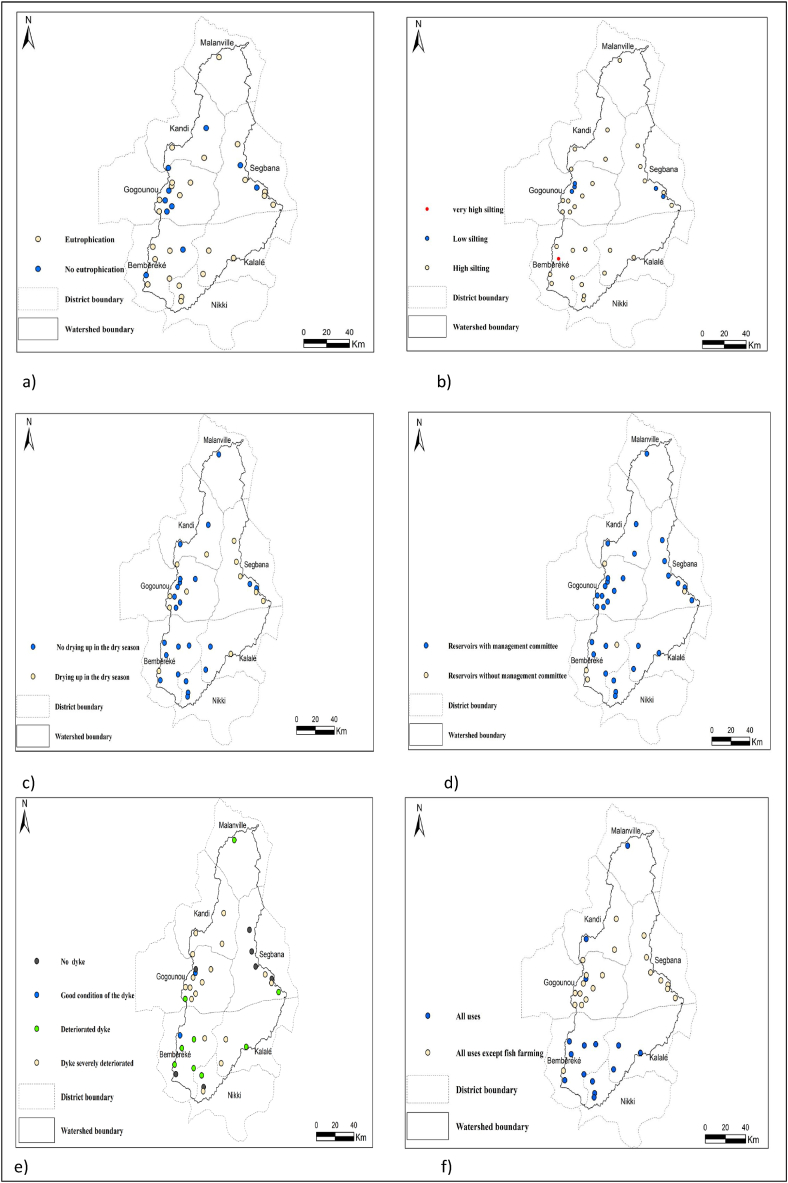
State of each reservoirs of the Sota catchment a) eutrophication state; b) siltation state; c) drying up state; d) presence or not of a management committee; e) dyke state; f) reservoirs uses.

Fig. 10 shows the distribution of the reservoirs affected by the potential deteriorating factors.

The analysis of Fig. 10 reveals that from the southern to the northern part of the Sota catchment the reservoirs are subjected to potential deteriorating factors. Siltation is observed at all the reservoirs of the Sota catchment within the district of, Bembereke, Kalale, Kandi and Nikki. Moreover, all the reservoirs of the Sota catchment within the district part of Kalale, Nikki, and Malanville are prone to eutrophication issue. Out of the 7 reservoirs in the district of Segbana, 5 reservoirs are subjected to drying up issue during the dry season. Out of the 11 reservoirs in the district of Gogounou, 6 reservoirs are subjected to eutrophication issue and 9 have deteriorated dykes. All the reservoirs of the Sota catchment within the district of Kandi have their dykes severely deteriorated. From a general view, the reservoirs in the Sota catchment area are in bad condition.

**Fig. 11:**
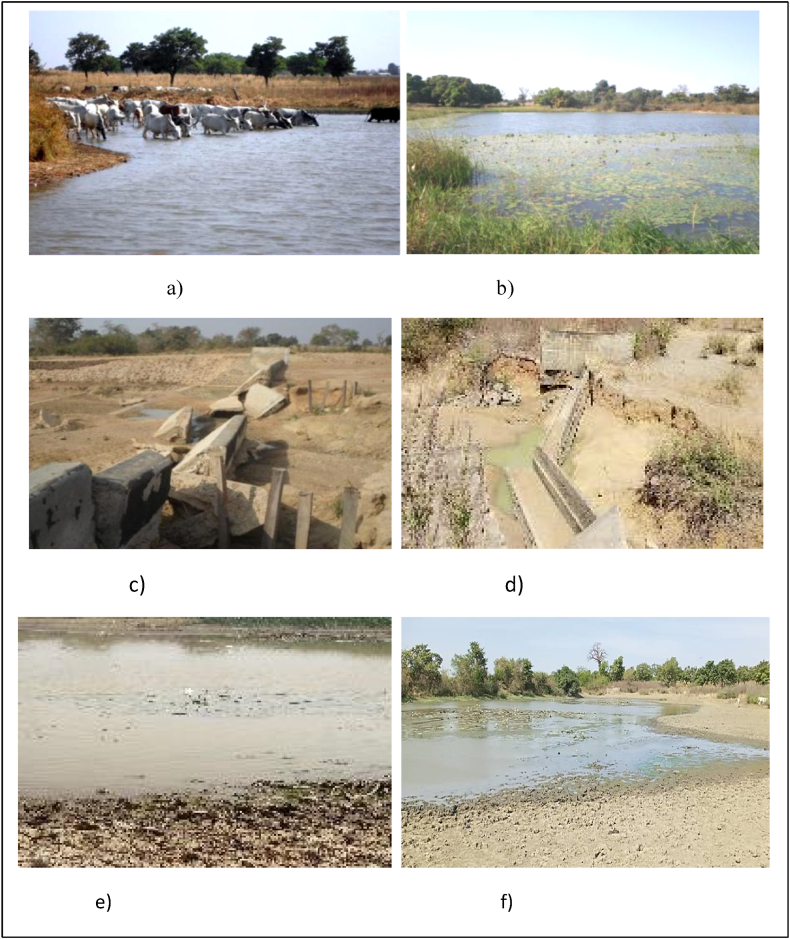
Pictures showing: (a) no waterers for animals, (b) eutrophication state, (c, d) broken spillway and dyke, (e, f) siltation state.

Fig. 11 shows some photographs related to the reservoirs state in support to Fig. 10.

## 4 Discussion

### 4.1 Reservoir density in the Sota catchment

Within the Sota catchment the reservoir density is 0.0026 km^−2^. This density is low compared to reservoir density detected in other basins in West Africa and some semi-arid regions. For instance, it is drawn from the studies of [11] that reservoir density is 0.0088 km^−2^ in the Nakambe River Basin in Burkina Faso, 0.0046 km^−2^ in the Mouhoun River Basin in Burkina Faso, 0.0031 km^−2^ in the Niger Basin in Burkina Faso. The reservoir density in the White Volta Sub-basin (Ghana) was 0.0051 km^−2^ in 2017 [36]. In the northern part of Brazil, high density reservoir network is noticed. For instance, the reservoirs density was 0.11 km^−2^ in 2011 in the Benguê catchment in Brazil [37] and 0.19 km ^−2^ in the Orós reservoir Basin [38]. Ref. [39] found a reservoir density of 1 km^−2^ in the watershed of Santa Cruz do Apodi in the semiarid region of Rio Grande do Norte, Brazil. The density of reservoirs in the Sota catchment is higher than that of the Comoe River Basin in Burkina Faso, which is 0.0016 km^−2^.

### 4.2 Factors associated with reservoirs' distribution in the Sota catchment

The results highlight that most of reservoirs within the whole district areas in the Sota catchment are located in the districts where livestock density and population density is high. This result is in accordance with the findings of [32] in the Nakambe River Basin in Burkina, [36] in the White Volta sub basin in Ghana, and Ref. [40] in the Upper East Region of Ghana. Ref. [36] pointed out that clusters of communities and high human and livestock population centers are the general determinants of reservoirs distribution in the White Volta Basin.

However a significant correlation has not been identified between reservoirs distribution and livestock density, population density and the respective average rainfall of the districts in the Sota catchment. This result corroborates the findings of [38] in the Orós Basin in Brazil who found that neither occupancy levels (rural population density plus animal density) nor rainfall significantly explain the spatial distribution of water reservoirs in that basin. But in the Sota catchment the small sample size of 7 observations (seven districts in the Sota catchment) used to perform the regression analysis may have affected the regression analysis results. Even if the same results are obtained with [38], this author performed the regression on a sample size of 26 observations (26 districts), much greater than ours.

The results highlight that the northern part of the Sota catchment prone to low rainfall amount, has the smallest number of reservoirs whereas the southern part of the catchment prone to high rainfall amount is home to the majority of the reservoirs. This result is not in accordance with the findings of [36] in the White Volta Sub basin in Ghana who found that the Upper East Region of the White Volta Sub basin having the lowest rainfall amount compared to North Region and the Upper West Region, has the highest number of reservoirs. In addition, Ref. [41] studying suitability of reservoirs' location based on Standard Precipitation Index, within the Bodri-Kuto River Basin in Indonesia, highlighted that the driest areas are very suitable for reservoirs locations. The relatively low prevalence of water reservoirs in the area prone to low rainfall in the Sota catchment, which seems abnormal, may be evidence that rainfall was not a significant factor dictating reservoirs' construction in the Sota catchment.

Regarding the geology, the sedimentary part of the Sota catchment where groundwater is easily accessible, is the least supplied with water reservoirs. This is in accordance with the findings of [38] at the Orós River Basin in Brazil. He argued that the semiarid sedimentary region of the Orós River Basin has a low number of water reservoirs compared to the crystalline region and then unlike the higher prevalence of reservoirs in areas with crystalline basement, the semiarid sedimentary regions have an alternative source of adequate freshwater, which is groundwater, for the rural populations. Groundwater reduces dependence on surface water sources and thus reduces the need for reservoir construction [38].

It appears that the low presence of reservoirs in the Sota region wouldn’t be due to a single factor nor to the studied (commonly cited) factors but also to others factors such as political orientation, transhumance highly prevailing in West Africa [19,42,43], and landform. The landform exerts influence on reservoirs distributions since the reservoirs are built following the natural landform in place where water can be easily stored more often in low slope areas. The influence of the landform is therefore captured by the catchment characteristics mainly the DEM (Fig. 2).

Most reservoirs in the Sota catchment are all ranked in the category of “small reservoirs”. Small reservoirs appear to be the main strategy to cope with water scarcity in rural areas. According to Refs. [44,45], the construction of a large number of small reservoirs enables access to water to a wide population, and their spatial distribution throughout most basins has increased due to their reduced cost, the availability of many favourable sites, and their easy access due to proximity.

The following authors have also notified the dominance of small reservoirs within some basins in West Africa and elsewhere [32] in the whole Burkina Faso territory, the northern part of Ivory Coast, Ref. [41] in the Bodri-Kuto River Basin in Indonesia, Ref. [37] in the Bengue catchment in the North-East Brazil, Ref. [1] in the Volta River Basin, and Ref. [40] in the Upper East Region in Ghana. For instance Ref. [1], reported that small reservoirs have been seen as important tool in achieving some of the goals of vision 2020 of Ghana and also the United Nations Millennium Development goals of poverty reduction.

### 4.3 Timeline of water reservoirs construction and their distribution

The temporal trend of the construction of the reservoirs in the Sota catchment indicates an increase of reservoir construction between 1992 and 1998. This can be largely attributed to droughts which occurred in the 1970s and 1980s (in many West African countries) and incited government funding for the construction of reservoirs. This result is in accordance with the findings of [32] who indicated that most of the reservoirs were built during the drought period (1974–1987) in the transboundary basins which across Burkina Faso (Nakambe River Basin, Comoe river Basin, Niger Basin and Mouhoun Basin). In the North Eastern part of Brazil, a semi-arid region, Ref. [46] also reported that most reservoirs were built during the drought period.

On the contrary, in the upper and middle Chattahoochee basins of the Georgia Piedmont (USA), Ref. [47] reported that the highest rates of reservoir construction occurred during subsequent suburban development between 1980 and 1990. The drought was not the trigger element for reservoirs construction in the upper and middle Chattahoochee basins. So, the reasons dictating the construction of water reservoirs may differ from one region to another. But the common remark that can be drawn is that in West Africa and in semi-arid region, reservoirs construction is mainly a way to adapt to climate change.

### 4.4 State of the reservoirs

The results revealed that the reservoirs are mainly subjected to the issues of siltation, eutrophication, inexistent waterers, and deteriorated dykes. These are the commons problems in many areas in West Africa where small reservoirs are neglected, not maintained and left at the care of the populations once built. These latter are not able to maintain the reservoirs and well manage them. Agricultural fields are even found at the proximity of reservoirs. The problem of negligence and degradation of the reservoirs (siltation, degraded dyke, poor management etc …) in Benin was also reported by Ref. [19]. In Burkina Faso, Ref. [48] argued that most of the small reservoirs in Burkina Faso are not maintained (deteriorated dyke), poorly equipped and there is a general lack of pastoral facilities (access tracks or corridors and drinking troughs) around these water reservoirs and the protection perimeters are not defined. Siltation, poor management, structural damages on dam body, leakage, seepage, were identified out of many others, as issues threatening reservoirs' sustainability in Trigray region in Ethiopia, and Mzingwane catchment in Zimbabwe, by the authors [49,50] respectively. For more precise information on reservoirs' eutrophication in the Sota catchment, laboratory analyses will make it possible to determine the rate of chemical contamination (nitrogen, ammonia, phosphorus) of the water. Yet most reservoirs of the Alibori (East Sota) catchment and eastern districts of the Sota catchment are found polluted in a recent study by Ref. [51]. With regard to the issue of siltation, direct measurement methods such as bathymetry will provide more accurate information on the siltation rate and as a result the volume of water remaining in the reservoirs for the various uses.

### 4.5 Implications for local development

This study calls for action for local development. More actions are needed to promote water reservoirs in the southern part and central western part of the Sota catchment where livestock density is high, livestock watering being the main purpose of the reservoirs. The districts being concerned are: Bembereke, Nikki, Kalale, Gogounou. However, the most important thing is to implement water management strategies for ensuring the maintenance and sustainability of the existing reservoirs. In fact, the analysis of reservoirs' state revealed poor maintenance of almost all the reservoirs in the Sota catchment. The reservoirs have been left to the community and managed by the management committee that has limited, or no capacity or resources to maintain them. Some reservoirs don’t even have a management committee. This situation doesn’t enable the reservoirs maintenance and requires a stronger involvement of the local authorities in the reservoirs management.

A dramatic situation noted is that, 70% of the reservoirs of the district of Segbana dried up in the dry season (Fig. 10). That calls for emergent actions such as reservoirs desiltation. In general, reservoirs desiltation is paramount since siltation is affecting the majority of the reservoirs in the Sota catchment and has downsides to their storage capacity and make them more vulnerable to early dry up. In fact, desiltation of reservoirs after decades of use could extend their lifespan significantly [52]. Furthermore, a strong and inclusive organization of reservoir stakeholders is imperative for ensuring reservoirs conservation and to set conditions to optimize their use and management in the Sota catchment so that the local populations and the municipality better benefit from the reservoirs. In addition, the implementation of reservoirs monitoring stations are required. Moreover, the implementation of agropastoral groundwater infrastructures such as agropastoral boreholes, agropastoral wells is to be promoted in order to supplement the water reservoirs subjected to many issues affecting their sustainability. Action that will combine groundwater and surface water management are highly desirable.

## 5 Conclusion

In short, this study has analyzed the spatial distribution and the state of water reservoirs in the Sota basin. Four parameters have been considered to deepen the analysis namely: livestock density, population density, rainfall distribution and geology of the Sota catchment. The results revealed that mostly small reservoirs contribute to water storage in the Sota catchment. Neither medium reservoirs nor large reservoirs have been identified in the catchment. The total capacity stored by all the reservoirs in the Sota catchment can be estimated at 2,060,844 m^3^. The reservoirs density of a value of 0.0026 km^−2^ is low compared to the reservoir density in the other basins in West Africa. In addition, neither livestock density, nor population density and rainfall explain the spatial distribution of water reservoirs in the Sota catchment. Of the two main geological (basement structures and sedimentary sandstone) structures, the basement structures are the most associated with reservoirs' location in the Sota catchment. The analysis of the reservoirs' state shows their poor state and management. Planning of reservoir construction needs to be included in the regional development plan to ensure equitable distribution in space and avoid high pressure of people and animals around some rare water points as reported by Ref. [11]. Further research investigating the other factors (e.g. transhumance, socioeconomic activities, and urbanisation) associated with reservoir planning, is required. A study on the hydrological impacts of the reservoirs on the Sota basin and climate change issues, is also required for a better management of the Sota catchment.

## Author contribution statement

Kevin S. Sambieni: Conceived and designed the experiments; Performed the experiments; Analyzed and interpreted the data; Contributed reagents, materials, analysis tools or data; Wrote the paper.

Fabien C.C. Hountondji: Conceived and designed the experiments; Performed the experiments; Analyzed and interpreted the data; Wrote the paper.

Luc O. Sintondji: Performed the experiments; Analyzed and interpreted the data; Wrote the paper.

Nicola Fohrer: Conceived and designed the experiments; Analyzed and interpreted the data; Wrote the paper.

## Funding statement

This work, forming part of a Ph.D. research project, was supported by the 10.13039/501100011853West African Science Service Center on Climate Change and Adapted Land Use (WASCAL), and the International Foundation for Science Grant number (I2-W-6673-1).

## Data availability statement

Data will be made available on request.

## Declaration of interest’s statement

The authors declare no conflict of interest.
